# Supplementary material for: The need for balanced dengue vaccine protection: Insights from Thai surveillance data on four serotypes
Source: PLoS Negl Trop Dis. 2026 May 22;20(5):e0014093. doi: 10.1371/journal.pntd.0014093 (PMC13196944; doi:10.1371/journal.pntd.0014093)
Supplement: S1 Table — Models compare DENV-2, DENV-3, and DENV-4 against DENV-1 (reference), adjusted for age (smooth term), secondary infection status, and calendar year (random effect). Odds ratios (OR) with 95% confidence intervals (CI) are shown for parametric terms. Effective degrees of freedom (edf) and p-values are shown for smooth terms. (DOCX) [file pntd.0014093.s001.docx]

**S1 Table. Generalized additive model results for serotype-specific associations.** Models compare DENV-2, DENV-3, and DENV-4 against DENV-1 (reference), adjusted for age (smooth term), secondary infection status, and calendar year (random effect). Odds ratios (OR) with 95% confidence intervals (CI) are shown for parametric terms. Effective degrees of freedom (edf) and p-values are shown for smooth terms.

| **Term** | **DENV-2 vs DENV-1** | **DENV-3 vs DENV-1** | **DENV-4 vs DENV-1** |
| --- | --- | --- | --- |
| **Parametric terms** | OR (95% CI), p | OR (95% CI), p | OR (95% CI), p |
| Infection type | 4.65 (2.95-7.35), <0.001 | 0.96 (0.65-1.42), 0.83 | 5.58 (2.81-11.1), <0.001 |
| **Smooth terms** | edf, p | edf, p | edf, p |
| Age | 2.43, <0.001 | 1.18, <0.001 | 1.59, <0.001 |
