# Supplementary figures and images for: The need for balanced dengue vaccine protection: Insights from Thai surveillance data on four serotypes
Source: PLoS Negl Trop Dis. 2026 May 22;20(5):e0014093. doi: 10.1371/journal.pntd.0014093 (PMC13196944; doi:10.1371/journal.pntd.0014093)

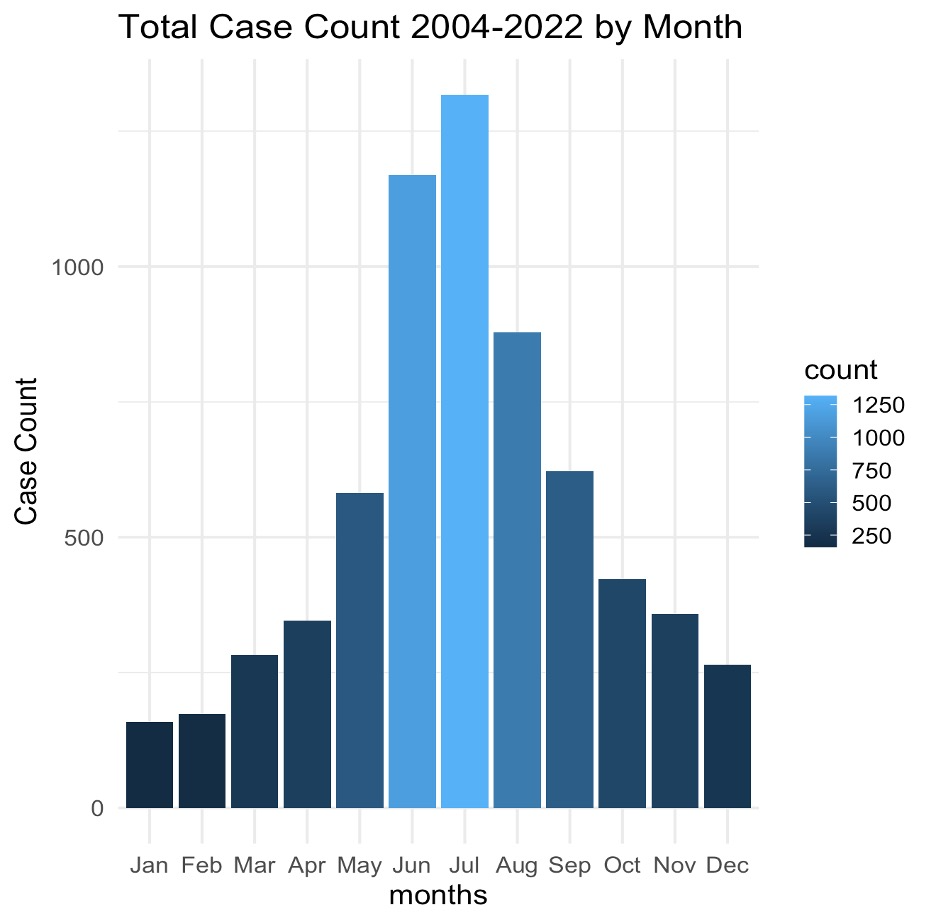

Supplement: S1 Fig — Infections peaked during the rainy season in Thailand (May-October). (TIFF) [file pntd.0014093.s002.tiff]
